# Tumescent Injections in Subcutaneous Pig Tissue Disperse Fluids Volumetrically and Maintain Elevated Local Concentrations of Additives for Several Hours, Suggesting a Treatment for Drug Resistant Wounds

**DOI:** 10.1007/s11095-020-2769-2

**Published:** 2020-02-10

**Authors:** John P. Koulakis, Joshua Rouch, Nhan Huynh, Holden H. Wu, James C. Y. Dunn, Seth Putterman

**Affiliations:** 1 Department of Physics and Astronomy, University of California Los Angeles, California, Los Angeles 90095 USA; 2 Department of Surgery, Division of Pediatric Surgery, University of California Los Angeles, California, Los Angeles 90095 USA; 3 Department of Radiological Sciences, University of California Los Angeles, California, Los Angeles 90095 USA; 4 Department of Surgery. Division of Pediatric Surgery, Stanford University School of Medicine, 300 Pasteur Drive, Alway M116, Stanford, CA 94305 USA

**Keywords:** antibiotic injections, antibiotic resistant wounds, chronic wounds, hydraulic permeability of subcutaneous tissue, pig subcutaneous tissue, subcutaneous injections, tumescent Injections, tumescent tissue

## Abstract

**Purpose:**

Bolus injection of fluid into subcutaneous tissue results in accumulation of fluid at the injection site. The fluid does not form a pool. Rather, the injection pressure forces the interstitial matrix to expand to accommodate the excess fluid in its volume, and the fluid becomes bound similar to that in a hydrogel. We seek to understand the properties and dynamics of externally tumesced (swollen) subcutaneous tissue as a first step in assessing whether tumescent antibiotic injections into wounds may provide a novel method of treatment.

**Methods:**

Subcutaneous injections of saline are performed in live and dead pigs and the physical properties (volume, expansion ratio, residence time, apparent diffusion constant) of the resulting fluid deposits are observed with diffusion-weighted magnetic resonance imaging, computed tomography, and 3D scanning.

**Results:**

Subcutaneous tissue can expand to a few times its initial volume to accommodate the injected fluid, which is dispersed thoroughly throughout the tumescent volume. The fluid spreads to peripheral unexpanded regions over the course of a few minutes, after which it remains in place for several hours. Eventually the circulation absorbs the excess fluid and the tissue returns to its original state.

**Conclusions:**

Given the evidence for dense fluid dispersal and several-hour residence time, a procedure is proposed whereby tumescent antibiotic injections are used to treat drug-resistant skin infections and chronic wounds that extend into the subcutaneous tissue. The procedure has the potential to effectively treat otherwise untreatable wounds by keeping drug concentrations above minimum inhibitory levels for extended lengths of time.

**Electronic supplementary material:**

The online version of this article (10.1007/s11095-020-2769-2) contains supplementary material, which is available to authorized users.

## Introduction

In this research article, we describe physics experiments on the in-vivo hydraulic permeability of subcutaneous pig tissue that has been tumesced (swelled) by an external injection. Our goal is to provide data that helps answer the question: can skin infections and chronic wounds be treated with targeted antibiotic injections? If concentrated antibiotic is infused into the tissue directly underneath an infected wound, will it cure the infection? The chief advantage of local treatment is that drugs can be delivered directly to the affected regions at higher concentration with reduced risk of systemic toxicity, making them particularly attractive for treating antibiotic-resistant infections [1]. Topical antimicrobial therapy for chronic wounds remains unproven despite tremendous effort in this direction [2]. We demonstrate below that tumescent injections into healthy subcutaneous tissue go beyond a surface treatment by infusing fluid in a way that causes it to completely permeate the tissue volume.

Antibiotic injections are not a standard method of wound care, and there is a void in the literature on the topic. One explanation, is that the procedure does not work, or that it is dangerous. For instance, some antibiotics may be toxic to tissue at elevated concentrations. Particularly worrisome is the risk of spreading the infection as the fluid disperses. Will the risk of spreading the injection be reduced if the fluid is a concentrated antibiotic? Even if the injection is safe in itself, if the antibiotic is absorbed rapidly by the circulation, the net effect of the injection is equivalent to a systemic dose, and the benefits of a local treatment are lost. We demonstrate below that fluid remains in the tumesced tissue near the injection site for many hours.

The medical questions which drive our physics-based experiments are motivated by the work of Jeffrey Klein and Barry Silberg. Jeffrey Klein invented the tumescent technique in the late 1980s as an alternative method of liposuction [3, 4], that has since proven itself a safe, low-complications procedure and become common practice [5]. Under this procedure, up to several liter of saline with additives are infused into subcutaneous tissue, causing it to swell and expand, before a suction cannula is inserted and fat is removed. Despite the trauma of the expansion, the tissue heals on its own [5]. Nowadays, the technique is used more broadly for cosmetic, vascular, orthopedic, and other procedures [6, 7]. Barry Silberg uses tumescent antibiotic injections supplemented by transcutaneous therapeutic ultrasound to treat soft skin and tissue pathologies [8]. The injection and ultrasound combination has successfully treated 94 patients under an institutional-review-board-approved phase 1 study [9], including many with drug-resistant infections. Despite injecting large volumes of fluid directly into and underneath infections, not a single case of infection spreading was reported. Tumescent antibiotic injections without externally applied ultrasound have not been reported.

Tumescent injections are performed by inserting a needle or cannula into subcutaneous tissue and infusing sufficient volumes of fluid at a fast enough rate to deliberately cause swelling [4]. This is distinguished from subcutaneous administration of biotherapeutics or antibiotics and hypodermoclysis (HDC), where care is taken to avoid swelling. HDC is becoming a popular method of hydrating geriatric patients due to its simplicity and safety, where liters of fluids, usually saline, are infused at a rate of about 1–2.5 mL min^−1^ for many hours [10–13]. These infusion rates are chosen to be slow enough that the infusate is dispersed before it can accumulate and cause swelling. Similar infusion rates are used when administering biotherapeutics or antibiotics subcutaneously (but not tumescently) as an alternative to the intravenous (IV) route [14]. As the non-tumescent subcutaneous route is used as an alternative for *systemic* delivery, research on it has focused on the altered pharmacokinetics, pharmacodynamics, and bioavailability [15–24], not on its potential for *localized* treatment. Tumescent infusions are done 10–100× faster, with total volumes ranging between 0.1–2 L, depending on the surface area to be covered.

This work investigates the properties and dynamics of the tumescent tissue subsequent to the injection. We use various methods to monitor the fluid infused into the healthy subcutaneous tissue of live and dead pigs, including diffusion-weighted magnetic resonance imaging (DW-MRI), computed tomography (CT), and 3D scanning. The use of animals was approved by the Animal Research Committee in accordance with accepted national standards and guidelines (Institutional Review Board Number 2014–142-03). Pigs used were part of an unrelated research project and no animal was sacrificed as part of this work. Our key findings are that 1) subcutaneous tissue can expand >3× to accommodate the fluid, which 2) becomes densely distributed throughout the volume of the subcutaneous tissue, and 3) remains localized for many hours. There are two distinct timescales of fluid dispersal: one is a localized spreading within the subcutaneous tissue itself that occurs over a few minutes, and the other is slow systemic absorption via the circulation over many hours. In light of these experimental insights, we end by proposing a hypothetical clinical procedure whereby tumescent injections of antibiotics are used to treat deep chronic wounds that extend past the dermis.

## Dilation of Subcutaneous Tissue

**Fig. 1:**
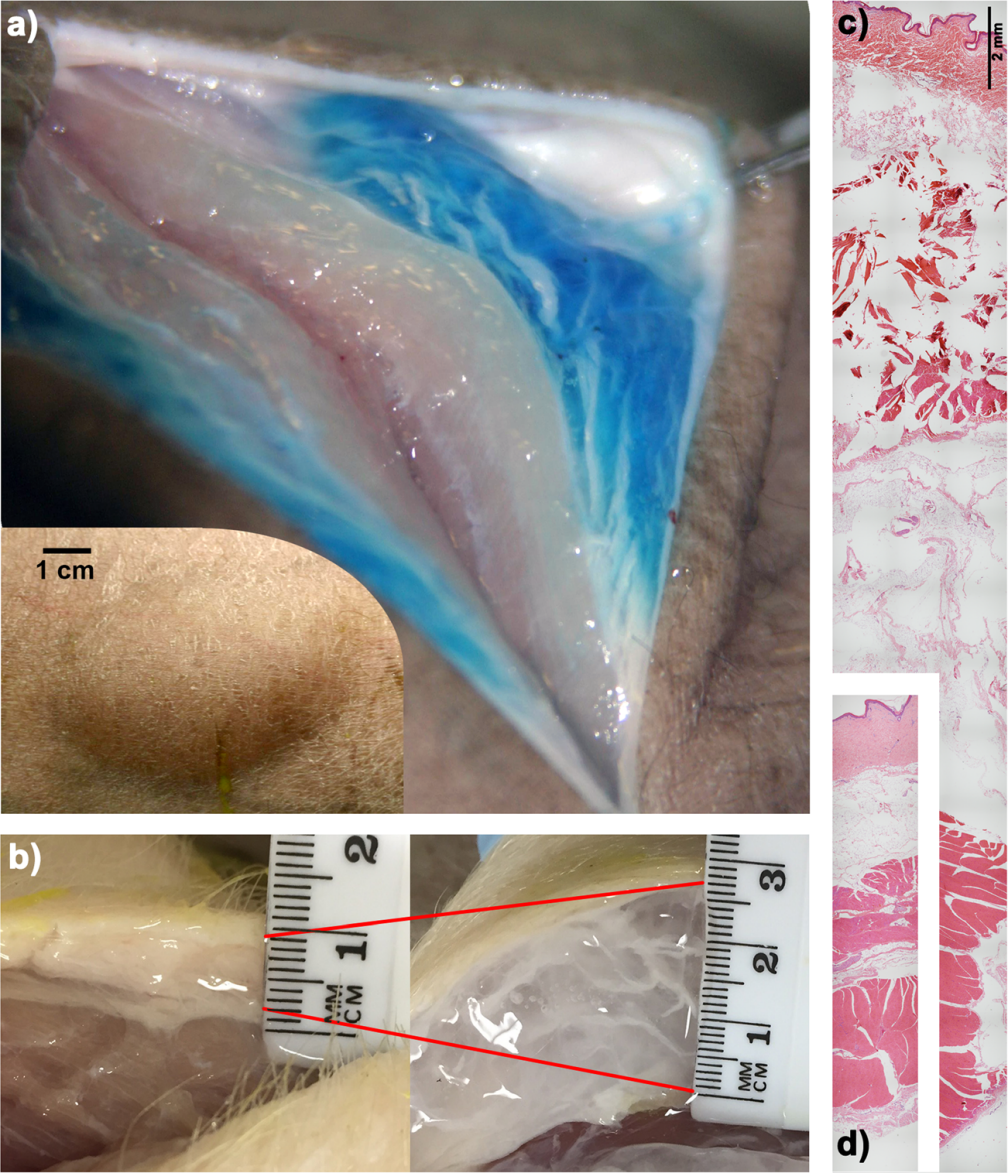
Tumescent injections in pig subcutaneous tissue. (a) Tumescent injection of 10 mL saline (dyed blue) causes a large, conspicuous bleb to form in the skin of a dead Yucatán pig, shown in the inset. Slicing into it reveals that the liquid is held in the subcutaneous tissue, which was forced to expand to accommodate the liquid. (b) Subcutaneous tissue of a Yorkshire pig swells in proportion to volume of injected saline, reaching about 4× expansion in this case. Histology of (d) normal and (c) tumescent subcutaneous tissue of a minipig after a 5 mL saline injection, shown at the same scale.

A 10 mL tumescent injection of blue-dyed saline into a dead Yucatán minipig leaves a bleb about 4 cm across, and 1 cm thick (Fig. 1a). Slicing into it allows visual confirmation of the dispersal density and quality. The fluid gets trapped within the extracellular matrix, and forces it to expand to several times its initial volume. Figure 1b shows an expansion of about 4× in the subcutaneous tissue of a dead Yorkshire pig, transforming it from soft and opaque, to translucent and firm. We know of no synthetic material of similar density that can dilate by such a large factor without cracking. The same experiment in gelatin, for instance, creates a crack whose leading edge propagates as more fluid is injected [25, 26].

Investigations of the fine structure of small volume (<0.5 mL) injection sites with X-rays [27] and cryomicrotome microscopy [28] have revealed that the fluid opens channels between adipose cells as it searches for the path of least resistance. These channels propagate and expand as more volume is infused in a process similar to the hydraulic fracture of rocks [29]. As the tumescent volume expands, the extracellular matrix stretches, opening up pores, and filling the extra volume with the infusate. Histological samples of tumesced and normal tissue (Figs. 1c and 1d respectively, see supplementary methods) reveal the microscopic disruption of the tissue after a 5 mL injection.

**Fig. 2:**
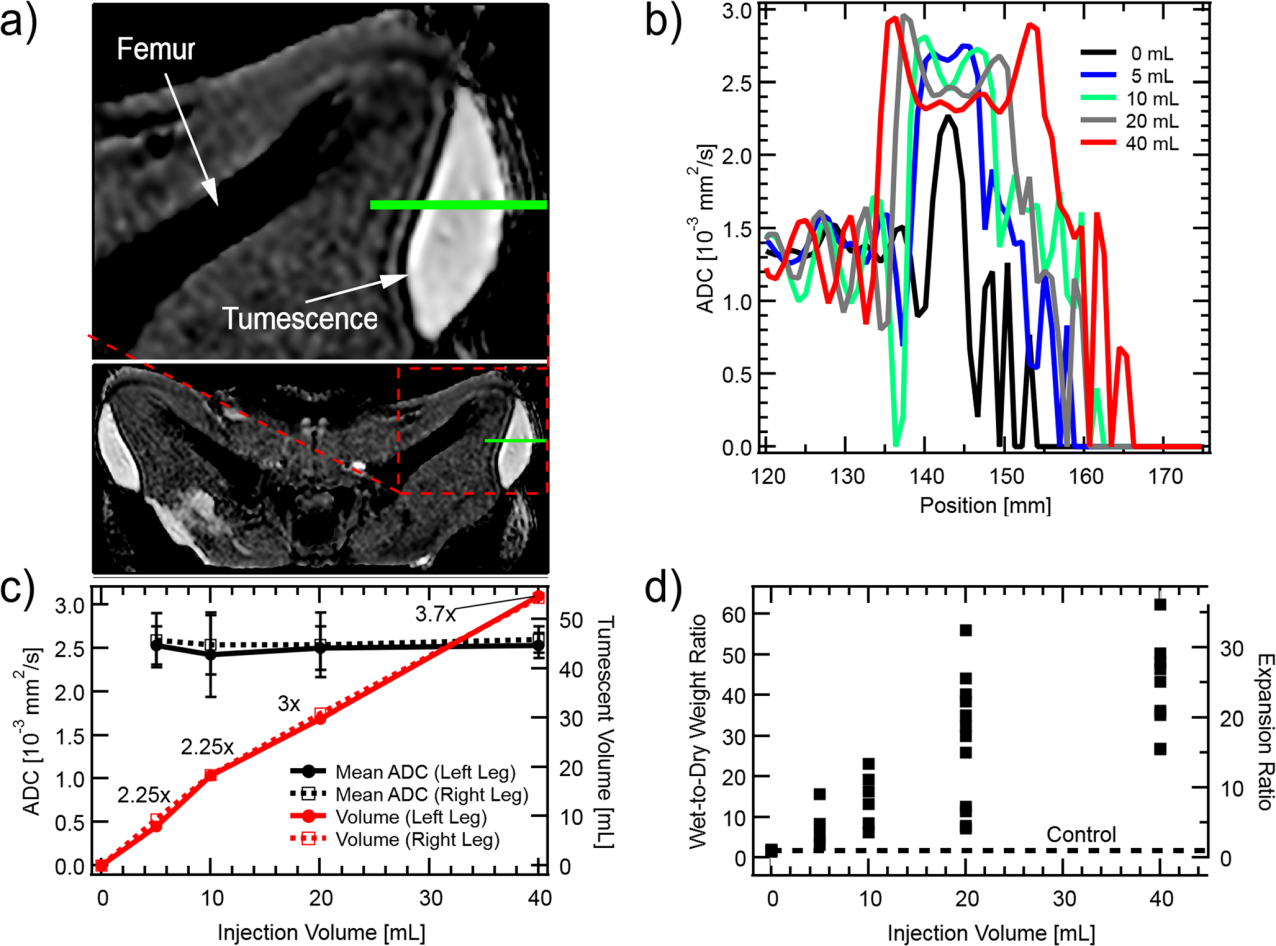
Expansion of tumescent tissue characterized by Diffusion-Weighted MRI. Panel a) is an apparent diffusion coefficient (ADC) map showing an axial cross section through the quadriceps of an adolescent Yucatán pig laying in a supine position after 40 mL physiological saline had been infused into the subcutaneous tissue of both right and left thighs. Whiter regions correspond to higher ADC. Lineouts along the green line in a) are shown in b), as more liquid is infused into the subcutaneous tissue. The tumescent volume and spatially averaged ADC are plotted in c). Uncertainty bars on the mean ADC plot indicate the standard deviation of the ADC within the tumescent volume. Numbers next to volume curve markers are the average expansion ratio as calculated from the tumescent volume. Expansion of subcutaneous tissue is also measured by surgically removing samples from within it, and weighing them before and after desiccation. Tumescent tissue samples were chosen to represent maximal levels of expansion (as in the bluer regions of Fig. 1a) - they are not randomly selected. The wet-to-dry weight ratio, and expansion ratio, are plotted in d). The scatter of the points with the same injection volume is characteristic of the spatial variations within the tumescent volume.

In the tumesced state, tissue permeability dramatically increases, enhancing fluid and additive dispersal. Indeed, the hydraulic conductivity (Darcy permeability) of normal subcutaneous tissue is low, about 10^−11^ cm^4^dyne^−1^sec^−1^ [30], but can increase by over four orders of magnitude upon swelling [31]. This property allows visualization of tumesced tissue with diffusion-weighted magnetic resonance imaging (DW-MRI). DW-MRI is a non-invasive method of measuring the apparent diffusion coefficient (ADC) - a measure of the molecular diffusion of water - within tissue [32]. We infused increasing volumes of saline (0–40 mL) into both right and left thighs of an anesthetized juvenile Yucatán pig, and DW-MRI was performed to quantify the tumescent volume and mean ADC as a function of total fluid volume infused (see supplementary methods). Figure 2a is an axial, ADC map intersecting the tumescent volume after 40 mL was infused. The ADC is about 1.4 ·10^−3^mm^2^s ^−1^ in the muscle underneath the tumescent tissue, as seen in lineouts crossing the tumescent region (Fig. 2b), consistent with the literature [33]. The ADC saturates at a value consistent with the self-diffusion coefficient of water, 2.5 ·10^−3^mm^2^s^−1^ [34], for even the smallest injection performed, 5 mL. It appears that even for such small injections, the tissue has expanded enough where the structure of the interstitial matrix negligibly restricts molecular diffusion. As more fluid is infused, the tumescent volume expands both into the empty space above the skin, but also into the body below; a small volume of normal, unexpanded tissue swells to a larger volume. Not shown, is that in addition to the thickness growing, the area covered broadens as well. We define the average expansion ratio to be the final tumescent volume divided by the initial, pre-expansion volume. The total volume of the tumescent region for a given volume injection gives a measure of the average degree of expansion (Fig. 2c). For instance, when 20 mL were infused, the tumescent volume was 30 mL, meaning that 10 mL of tissue expanded to 30 mL, and so the average degree of expansion was 3× [28].

However, there is considerable variation in the degree of expansion within the tumescent volume, as indicated by the visible white streaks within the blue region in Fig. 1a. To characterize the amount of variation, small samples (about 0.5 g each) were cut from the tumescent region, and weighed before and after desiccation (see supplementary methods). While taking samples, we attempted to avoid regions with visible white streaks and focused instead on the more transparent, high-expansion regions. The streaks were expanded as well, but to a lesser degree. Consequently, the resulting data should be interpreted as representative of local maxima in expansion, not random samples. For the smaller volume injections, it was difficult to avoid the streaks embedded within the tumescent volume and more found their way into the samples. The wet-to-dry-weight-ratio is shown in Fig. 2d for various volumes injected, and is roughly proportional to volume injected. Control samples, taken from regions without injection, have a wet-to-dry-weight-ratio of 1.7, while tissue tumesced with 20 mL saline had regions with ratios of 10–50, which implies an expansion of 6–30×.

## Local Spreading within the Interstitial Matrix Occurs over a Few Minutes

We observe distinct processes as the infused fluid dissipates and the tissue returns to normal. Once the injection is complete, a pressure gradient exists between the tumesced site and the surrounding tissue that drives outwards flow confined within the subcutaneous tissue. As the flowing fluid overtakes more unexpanded tissue, the tumescent volume increases by the amount of tissue overtaken and softens because the injected fluid is spread over more tissue. This process occurs over a few minutes and is observed in both live and dead pigs. In live pigs, we also observe the circulation absorbing the fluid and distributing it through the body on a multi-hour timescale, as discussed in the next section.

**Fig. 3:**
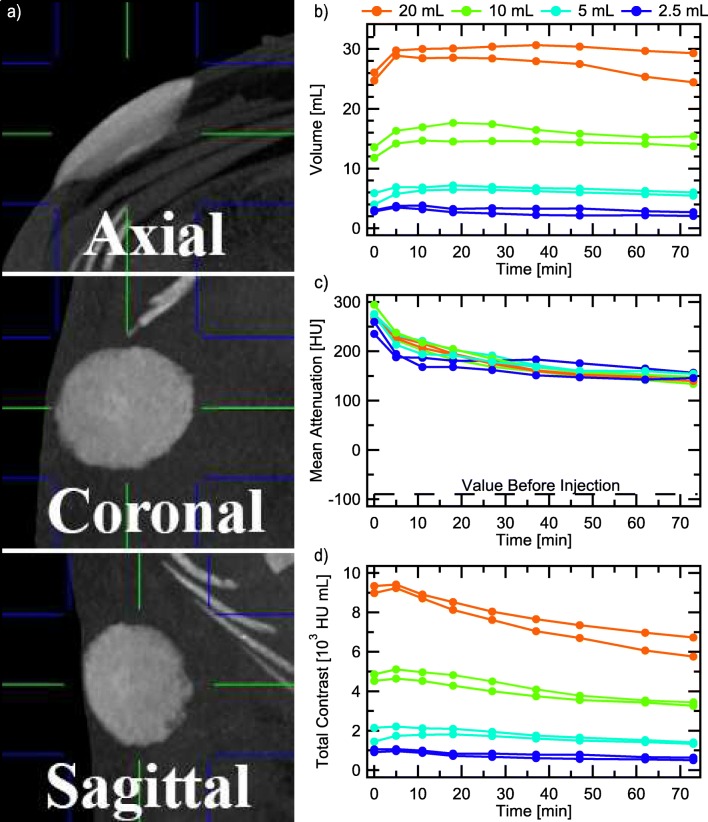
Evolution of various volume tumescent injections over an hour as measured by CT. Panel (a) shows three orthogonal CT views of a 20 mL tumescent injection into the abdomen of a live, adult Yucatán pig, viewed in maximum-intensity-projection (MIP) mode. Green and blue lines indicate the center and extent of the MIP slabs. The volume (b), and spatially averaged attenuation (c), are multiplied to produce the total contrast curve in (d).

CT imaging allows tracking the 3D tumescent region over time. Eight tumescent injections were made in the abdomen of an anesthetized adult Yucatán pig, 2 each of 2.5, 5, 10, and 20 mL physiological saline solution containing dilute iodine contrast and CT scans were taken every 5–10 min for 70 min (see supplementary methods). Figure 3a displays three orthogonal views of a 20 mL injection immediately after the injection. Because the contrast increased the x-ray absorption of the tumescent fluid, the tumescent region is easily discerned in the image. The volume and mean attenuation within the tumesced regions were measured, and are displayed in Figs. 3b and 3c respectively. Note that there is a rise in the tumescent volume during the first 5–10 min, corresponding with a drop in the mean attenuation, despite no additional fluid being infused. This reflects the fluid spreading within the subcutaneous tissue, overtaking more unexpanded tissue, but staying localized (not removed by lymph and/or blood circulation [35]). Multiplying the mean attenuation by the volume gives the total contrast curve in Fig. 3d, which is a measure of the total amount of contrast near the injection site. If the contrast was being taken away from the tumesced region by the circulation, this curve would decrease, and so its initial flatness confirms our localized spreading interpretation. On longer timescales (discussed below), we do see absorption by the circulation, as indicated by the slow decrease in the total contrast curve. Once again we can estimate the average expansion by comparing the mean attenuation within the tumesced tissue (200–300 HU depending on the time after injection) to that in normal subcutaneous tissue (−90 HU) and that in the pure solution (500 HU), yielding average expansions of 2–3×, consistent with our previous estimates.

**Fig. 4:**
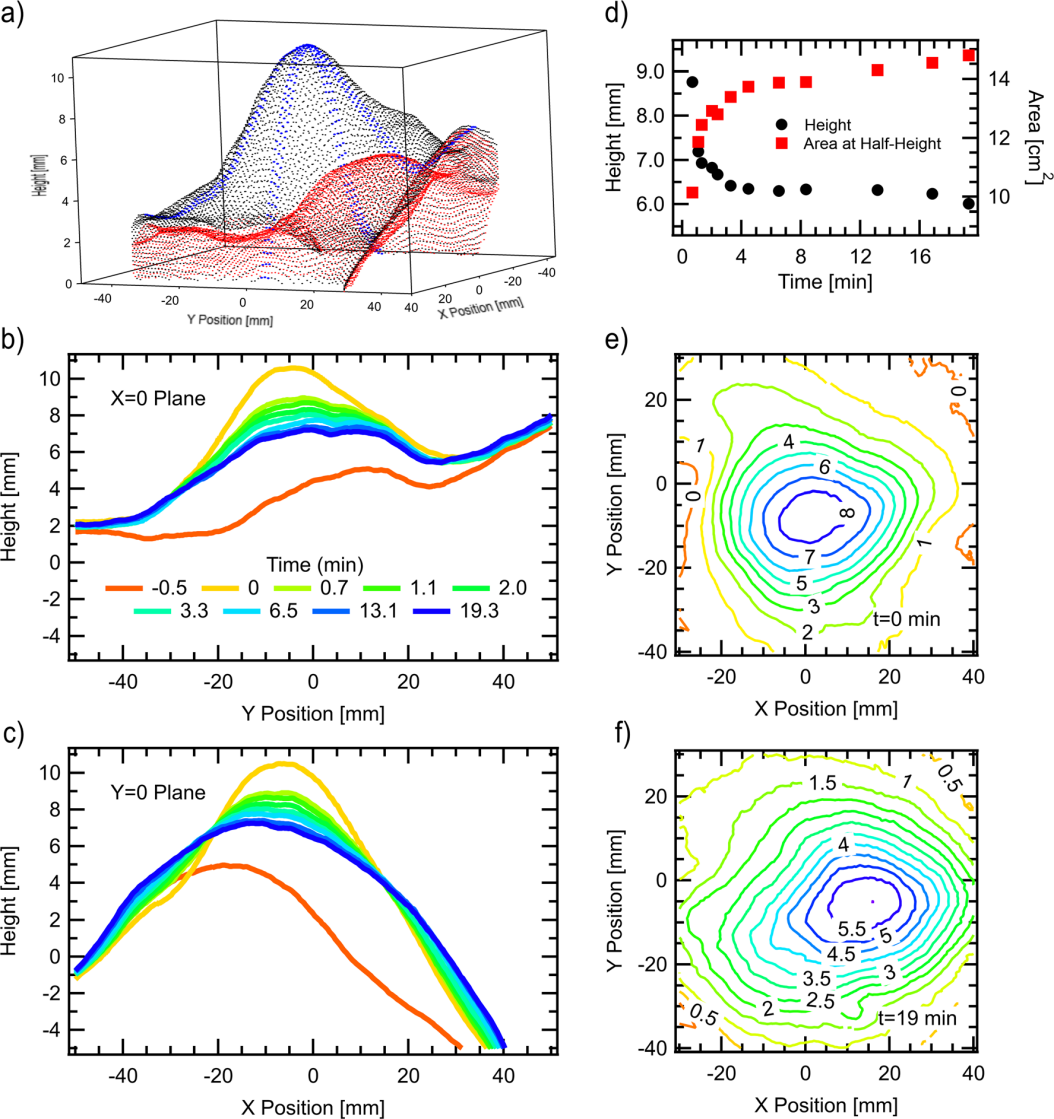
3D scans yield the tumescent skin profile over time. Panels (b) and (c) are lineouts of the full 3D data, shown immediately before (red) and after (black) a 20 mL injection in panel a). Dots in two perpendicular bands centered on *X* or *Y* = 0 along the tumescent surface in (a) are shown in blue to help guide the eye. The difference between the skin surface at various times after the injection, with that before the injection, is used to calculated the tumescent height and cross-sectional area at half-height plotted in (d). Panels e) and (f) are contour plots of the difference at times t = 0 and t = 19 min respectively. The process shown here is the flow within the interstitial matrix immediately following injection, which results in a dense distribution of the injected fluid and additives throughout the tumescent volume.

We were able to isolate the localized spreading dynamics from the systemic absorption by studying the evolution of the tumescent skin profile in a dead pig, where there is no circulation to distribute the injection through the body. A 20 mL tumescent injection was performed in the abdomen of a dead juvenile Yucatán, and 3D photographs were taken with a Fuel-3D Scannify system periodically for 20 min to quantify the shape of the bleb on the skin surface and its motion over time (see supplementary methods). Figure 4a shows the raw data before and immediately after injection. Note that the Z-scale is 10× smaller than the X and Y scale. Lineouts along X = 0 and Y = 0 are shown in Figs. 4b and 4c respectively for various times after the injection. It is clear that the tumesced volume changes shape very rapidly early on, dropping more in the first two minutes than in the subsequent seventeen. After about 10 min, the changes are imperceptible. The contour maps of Figs. 4e and 4f show the difference between the skin profile before the injection and that at t = 0 or t = 19 min respectively. Plotting the max difference (height) and cross-sectional area at half-height over time gives Fig. 4d. The product of the height and area curves (not shown), a quantity proportional to the fluid volume injected, is constant within uncertainties, indicating that no substantial fraction of the fluid was removed from the injection site by systemic circulation. In a live pig, when the local spreading slows enough, the circulation becomes the dominant mechanism of further distribution.

There is an open theoretical question of the expected form of such a decay based on Darcy’s law in a medium whose permeability depends on the degree of expansion. Poroelastic models that couple flow and gel mechanics [36–39] need to include nonlinear effects such as the reduced Darcy resistance in expanded regions if they seek to accurately capture the observed dynamics. With nonlinear modeling, it may be possible to back out the intrinsic permeability of subcutaneous tissue [37] from data such as that presented in Fig. 4.

## Systemic Absorption Occurs over Several Hours

Eventually the tumescent fluid dissipates and the interstitial tissue returns to normal. This work is the first to measure the dissipation timescale directly. Indirect estimates based on plasma lidocaine concentrations following tumescent liposuction [40] suggest that some infused fluid remains localized for ~10 hr. But tumescent fluid used for anesthesia usually contains dilute epinephrine, a vasoconstrictor, to minimize blood loss and slow down the rate of lidocaine absorption [41]. We expect epinephrine would also extend the fluid residence time.

We performed CT scans periodically over seven hours to observe the fluid dissipation both with and without epinephrine. As before, eight tumescent injections were made in the abdomen of an anesthetized adult Yucatán pig, 2 each of 2.5, 5, 10, and 20 mL physiological saline solution containing dilute iodine contrast. A few weeks later, we performed the same experiment on the same pig, with the addition of 1:100000 epinephrine to the tumescent solution (see supplementary methods).

**Fig. 5:**
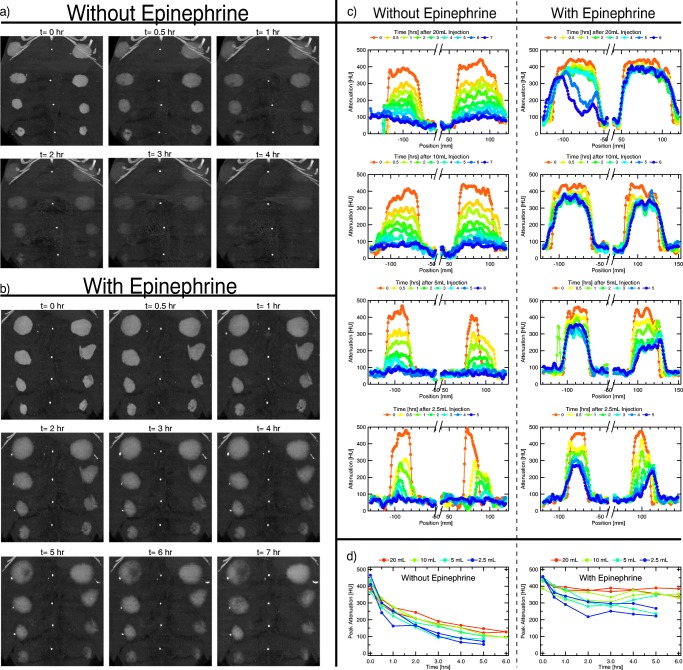
CT coronal images in MIP mode showing the time evolution of various volume tumescent injections of saline with iodine contrast into abdominal subcutaneous tissue of an adult Yucatán pig, both without a), and with b), 1:100000 epinephrine. Two injections each of 20 mL, 10 mL, 5 mL, and 2.5 mL were done on left and right sides of the pig’s abdomen, with large volume injections closer to the pig’s head (top of images) and smaller ones towards the feet (bottom of images). Images for *t* > 4 hr. in (a) are not shown because the injections are difficult to see. The white dots along the center of the abdomen are markers placed in the same axial plane as the injection location. White dots that form on the side of the tumescent spots in (b) are tumescent fluid that leaked out of the puncture wounds. Lineouts crossing the center of the tumescent spots of a) and b) are shown in panel (c). Panel (d) shows the peak of the lineouts over time, indicating the return of the tumescent tissue to normal. Epinephrine dramatically increases the fluid residence time in the tumesced tissue.

Coronal images in maximum-intensity-projection (MIP) mode are shown in Figs. 5a and 5b without and with epinephrine respectively. In both cases, localized spreading occurs between the *t* = 0 and the *t* = 0.5 hr images, as indicated by the boundary of the tumescent region becoming less sharp; epinephrine did not affect this. As time progresses, the contrast fades away. Without epinephrine, it is hard to see any remaining contrast after three hours. With epinephrine, it is easily visible after seven hours. For a more precise comparison, we took lineouts crossing the center of each spot, seen in Fig. 5c, and plotted the peak value vs time in Fig. 5d. Attenuation levels after six hours in the presence of epinephrine are comparable to those after half an hour without epinephrine. However, even without epinephrine, the contrast remains substantially localized for 2–3 hr for the larger injections. In general, the larger injections decay slower.

## Applications to Chronic Wounds, Systemic Drug Delivery

This work observed the localization and spreading of saline, with contrast (CT), and without contrast (MRI, 3D scanning). The data show that tumescent injections force the expansion of subcutaneous tissue from the inside out, dilating the interstitial matrix, and saturating it with fluid. Average expansion ratios of 2 − 4× are readily achievable, a factor high enough whereby the hydraulic resistance of the interstitial matrix is greatly reduced. The fluid residence time is 2–3 hr without any special preparation, and can be extended to well over 7 hr with the use of epinephrine. Systemic circulation eventually spreads the fluid to the entire body and the injection site returns to normal. Although the spreading of drugs added to the saline may be more complex due to, for example, cellular uptake, changes in stability, and degradation, and will require further study on a case-by-case basis, some general conclusions may be drawn regarding the expected dynamics of additives. Those that are small and stay in solution are expected to spread in a similar manner to the saline. Larger additives whose dimensions are comparable to or larger than the expanded matrix pore size will be preferentially filtered and remain localized longer. Take for example, the iohexol molecule used as a contrast agent in the CT experiments. The uniformity of the contrast in the tumescent volumes pictured in Figs. 3 and 5, in particular the lack of iodine accumulation near the injection site, suggests that the interstitial matrix, and/or cellular uptake [35], does not measurably restrict its motion and that it was carried with the saline. Since the dimesions of the iohexol molecule are comparable to those of the antibiotic Cefazolin, we expect Cefazolin to spread similarly. Based on these findings, we propose treating skin infections or chronic wounds that extend into subcutaneous tissue with targeted antibiotic injections directly into the affected tissue.

Chronic wounds are by definition those which have not responded to traditional therapy and last for prolonged lengths of time. The most common types fall into four categories: diabetic ulcers, pressure ulcers, venous ulcers, and arterial ulcers. One hallmark of most chronic wounds is persistent, runaway inflammation in which growth factors, cytokines, immune cells, and other healing mediators are locked in an imbalanced state that prevents healing [42–46]. Prolonged attempts of wound closure lead to fibrin cuffs surrounding capillaries that hinder cell migration, and the flow of nutrients, healing mediators, and oxygen and to surrounding tissue [47–50]. Infections, often resistant to antibiotics, contribute to the bioburden and non-healing of wounds.

In choosing antibiotic dosage, there is a trade off between minimizing systemic toxicity and effectively treating the infection. This balance can be optimized by keeping drug concentrations high at the infection only, which is what tumescent antibiotic injections can provide. By keeping concentrations low where they are not needed, systemic load is minimized. With this technique, we estimate that drug concentrations at the injection site can be increased by roughly the volume ratio of the infection to the rest of the body (≤1000×) compared to systemic delivery of the same dose, and infections that were otherwise untreatable might become accessible. Antibiotics that are likely to benefit from targeted delivery are those whose minimum-inhibitory-concentration (MIC) is high enough where toxicity is a concern if given systemically, but low enough where the MIC can be reached with targeted injections. For example, Methicillin-resistant *Staphylococcus aureus* (MRSA) is an important pathogen commonly found on both acute and chronic wounds [51, 52]. Silberg and Nicolau [1] have demonstrated that nearly all of 1239 strains of MRSA tested succumbed to Cefazolin at 512 mg L^−1^. Comparable concentrations are achievable and tolerated [9, 19] with targeted injections. An important line of research will be to identify the MIC of drug-resistant organisms to various antibiotics to best plan treatment regiments.

Complicating factors in the treatment of chronic wounds include the presence of biofilms, which are present in 60% of chronic wounds, but only 10% of acute wounds [53]. When the bacterial density in a wound exceeds a threshold, bacteria that are otherwise planktonic and mobile form polymicrobial colonies and begin to act collectively [54]. Bacteria sense that their density has reached critical through quorum sensing and begin to express different genes [54]. They excrete exo-polymeric substance, which forms the biofilm to house the microbes [54]. The biofilm contains structures such as water channels that bring in nutrients and carry away waste, and provides a barrier to antimicrobial agents and immune cells [54]. Microbes in the biofilm become sessile and reduce their metabolism, which reduces their susceptibility to antibiotics [54].

Tumescent antibiotic injections, when performed in conjunction with sharp debridement and skin grafting as needed, might be a safe and effective approach to treating deep skin and soft-tissue infections, particularly chronic wounds, and especially antibiotic-resistant ones. The volumetric expansion of the affected tissue simultaneously addresses several core issues of chronic wounds. It is the basis of 1) a dense distribution of antibiotic to infected tissue that is often inaccessible to systemic dosing, 2) a dramatic increase in the hydraulic permeability and effective macromolecule diffusion constant in regions with typically poor circulation, and 3) a fluid and additive residence times of several hours.

Beyond keeping antibiotic concentrations high for a given time duration, there may be other mechanisms by which tumescent injections promote chronic wound healing. Further experiments will probe whether the large volume infusions and subsequent absorption also act to 4) structurally and chemically disrupt biofilm, and/or 5) flush the imbalanced inflammation mediators that result in the chronicity of the wound and thereby reset the wound to an acute-like state. Biofilm may not be as resilient to a 2 − 4× volume expansion as the extracellular matrix is, resulting in structural damage to the film itself. The phenomenon of quorum sensing may be disrupted by the saline flush as well as the increased average interbacterial distance during the expansion, encouraging bacteria to revert back to their planktonic state. As discussed, wounds become chronic when natural healing mechanisms go awry and become stuck in an imbalanced state. Flooding the wound volume with fluid will necessarily dilute any inflammation and healing mediators and profoundly alter the local microcirculation. Imbalanced mediators are flushed away through the systemic absorption of the tumescent fluid via the expanded interstitial network and circulation, and when the tissue returns to its unexpanded state, the working point of many nonlinear healing processes will be different. The flushing strongly disturbs the balance of healing and inflammation mediators and may act to reset the stuck, chronic healing process to a new, healing-promoting state.

On the question of time duration required for concentrated antibiotic to eradicate an infection, we offer some comments. Since bacteriostatic drugs work by preventing the growth of bacteria, we can estimate that that drug should remain concentrated for several bacteria division times. Doubling times during the exponential-growth phase are 20–40 min for most MRSA and other common strains [55, 56], and so a residence time of a few hours satisfies this requirement. Bacteria in chronic wounds, however, might not be in the exponential-growth phase, so longer times may be needed. On the other hand, bacteriostatic drugs often become bactericidal at high concentrations [57], whereupon the doubling-time-scale is no longer applicable. Further studies are needed to provide a definitive answer.

As mentioned in the introduction, Silberg uses tumescent antibiotic injections (4 mg mL^−1^ Cefazolin [9]) supplemented with transcutaneous application of therapeutic ultrasound at 3 W cm^−2^ to successfully treat chronic wounds, many of which have antibiotic resistant infections. Therapeutic ultrasound has recently been shown be ineffective at accelerating tumescent fluid dispersal [58] or causing cavitation in tumescent *healthy* tissue [59]. It may be that ultrasound is essential to disperse the drug in *inflamed* tissue, enhance organism antibiotic susceptibility [60–62], or for some other reason. However, given the physical picture we present, there is a clinical possibility that the tumescent antibiotic injection is effective on its own.

Our findings also have implications for systemic drug delivery. The pharmacokinetics of subcutaneously administered drugs may be tailored by controlling injection volume and vasoconstrictor concentration to form a tumescent drug depot that is slowly released. When delivering large drugs such as monoclonal antibodies, injection sites may be tumescently primed to pre-expand the matrix before following with the drug to prevent filtration and concentration of the large molecule near the needle. Although we did not investigate the use of hyaluronidase to facilitate the spreading of tumescent injections [63], there is an opportunity to combine the techniques to achieve more complex dynamics.

The ability of subcutaneous tissue to expand to a few times its normal volume and recover safely [5] has been underappreciated by the medical community. We hope that by distilling the unique properties and dynamics of the tumescent state, this work stimulates discussion and focuses effort to fully realize its therapeutic implications.

### **ACKNOWLEDGMENTS AND DISCLOSURES**

We gratefully acknowledge support from the Paul S. Veneklasen Research Foundation. We thank the reviewers for feedback which improved the manuscript; Novena Rangwala, Ben Wu, Chase Linsley, Barry Silberg, and David Nicolau for helpful discussions; and Genia Dubrovsky, Elvin Chiang, Adora Samaan, and the UCLA DLAM and TRIC lab staff for assistance. The regents of the University of California have submitted a patent on this work. J.K, J.R., N.H., J.D., and S.P. are inventors. S.P. has an ownership stake in Sonescence, Inc.

## Electronic supplementary material

ESM 1 Supplementary Methods (DOCX 18.3 kb)

## Data Availability

The datasets generated during the current study are available in the Dryad repository, 10.5068/D14H3M.
