# Supplementary material for: Tumescent Injections in Subcutaneous Pig Tissue Disperse Fluids Volumetrically and Maintain Elevated Local Concentrations of Additives for Several Hours, Suggesting a Treatment for Drug Resistant Wounds
Source: Pharm Res. 2020 Feb 10;37(3):51. doi: 10.1007/s11095-020-2769-2 (PMC7010616; doi:10.1007/s11095-020-2769-2)
Supplement: Supplementary file 1 — Supplementary Methods (DOCX 18.3 kb) [file 11095_2020_2769_MOESM1_ESM.docx]

## 9 Supplementary Methods

### 9.1 Pigs

The use of animals was approved by the Animal Research Committee in accordance with accepted national standards and guidelines (Institutional Review Board Number 2014-142-03). All materials were FDA approved for use in humans. Female juvenile mini-Yucatan pigs weighing at least 5 kg were purchased from S&S Farms (Ramona, CA). Pigs were housed in several animal care facilities in compliance with NIH requirements. Animals were part of another research study that required a laparotomy. Efforts were made to avoid the area of previous abdominal incision for tumescent injections. Tumescent injections were either performed on live or dead animals. Dead pigs were obtained from the laparotomy research study within one hour of euthanasia. For the live animals, animals were intubated, anesthetized with inhaled oxygen, and vaporized isoflurane for the tumescent injections. All live animal procedures were survival studies.

### 9.2 Tumescent Injections

A detailed description of the tumescent technique is given in [31]. Tumescent injections of physiological saline into subcutaneous tissue were performed using an 18 or 20 gauge needle. The needle was held in place until the full volume (between 2.5-40 mL ) was injected over 10-30 s. Methylene blue was used as the dye in Fig 1a.

### 9.3 Histology

Samples of normal and tumescent (5 mL injection) subcutaneous tissue were acquired from a dead, juvenile Yucatán pig. Fixed tissue was aligned in perpendicular cross sections and embedded in paraffin. Tissue blocks were cut into 5 $\mu$m sections and stained with hematoxylin and eosin. Sections were examined and imaged at 4x magnification using light microscopy (Olympus Corporation, Waltham, MA).

### 9.4 Diffusion-Weighted MRI

MRI scanning was performed in the UCLA Translational Research Imaging Center (TRIC) on a 3 T whole-body system (MAGNETOM Prisma, Siemens, Erlangen, Germany) and a body array coil. Diffusion-weighted images were acquired with 30 slices, 5 mm slice thickness, $1\times1$ mm^2^ in-plane spatial resolution, 5 b values (0, 50, 100, 400, 800 s mm^-2^), and 3 signal averages. One complete DW-MRI dataset required 3 min of scan time. The apparent diffusion coefficient (ADC) was calculated by the scanner software using a mono-exponential isotropic Gaussian diffusion model.

Physiological saline was infused into the subcutaneous tissue of both right and left thighs of an adolescent Yucatán pig. Before infusion began, $T_{1}$-weighted and $T_{2}$-weighted images were taken to help localize the diffusion-weighted images. Diffusion-weighted images were acquired after total infusions of 0, 5, 10, 20, and 40 mL in the same region (for example an additional 5 mL were infused after the first 5 mL to get a total volume of 10 mL, etc.). Due to the long scan time required for the diffusion-weighted dataset, breath holds could not be administered. Despite choosing regions on the legs which were relatively stable, some motion artifact does appear.

Images were analyzed with OsiriX Lite DICOM viewer and analysis software. An example diffusion-weighted image can be seen in Fig 2a. Lineouts of the ADC were taken at the same axial slice through a location central to the tumescent volume, and shown in 1b. To verify that the lineouts for different injection volumes were properly aligned, we extended them to cross the femur, a readily identifiable feature. The drop in ADC at the femur location occurred at the same coordinate for each lineout. To measure the volume and mean ADC within the tumesced tissue, a region of interest surrounding it was selected using the 3D grow region tool with a “confidence” algorithm (parameters were adjusted by hand until the selected region was a good match to tumescent volume but were in the range: 2.5-3 multiplier, 5 iterations, 1-2 pixel initial radius). The volume and mean ADC within the regions of interest are plotted in Fig 2c.

### 9.5 Wet-to-Dry Weight Ratio

After tumescent injection into the subcutaneous tissue of a dead juvenile Yucatán pig, the tumescent tissue was surgically removed. Small samples, weighing about 0.5 g , were cut from it, and placed in a small ziplock bag. We took 6 samples with 0 mL (control) injections, 4 samples with 5 mL injections, 6 samples with 10 mL injections, 12 samples with 20 mL injections, and 9 samples with 40 mL injections. The ziplock bags were weighed empty, and with a fresh sample. Surgically removing tumescent tissue, cutting small samples, and placing them into the bag was done quickly, to minimize the volume of liquid coming out of the samples; after just a few minutes of sitting in the bag, a considerable fraction of the liquid initially contained within the sample had come out (but was accounted for in the weight since it was already contained in the bag). Bags containing the samples were kept open and upright in a vacuum chamber until the samples were fully desiccated. Afterward, the dry samples were weighed together with their bag.

Expansion ratios were calculated from wet-to-dry weight ratios as follows. The wet-to-dry ratio of control samples is,

$R_{c}=\frac{f\rho_{w}+(1-f)\rho_{d}}{(1-f)\rho_{d}},$ (1)

where $f$ is the water volume fraction in unexpanded tissue, $\rho_{w}$ is the density of water, and $\rho_{d}$ is the average density of the “dry” tissue. Experimentally we find that $R_{c}=1.7$ (Fig 2d). Upon injection of fluid, a volume of unexpanded tissue, $V_{unexp}$, increases in volume by an amount $V_{extra}$ that is due purely to additional saline (assumed to be at water density with negligible error). Its wet and dry mass ($m_{w}$ and $m_{d}$) are therefore,

$m_{w}=V_{extra}\rho_{w}+V_{unexp}(f\rho_{w}+(1-f)\rho_{d}), and$ (2)

$m_{d}=V_{unexp}(1-f)\rho_{d}.$ (3)

with ratio,

$R_{s}=\frac{m_{w}}{m_{d}}=R_{c}+\frac{V_{extra}}{V_{unexp}}\frac{\rho_{w}}{(1-f)\rho_{d}}.$ (4)

We define the expansion ratio to be,

$ExpansionRatio=\frac{V_{extra}+V_{unexp}}{V_{unexp}}$ (5)

$=1+(R_{s}-R_{c}) \frac{\rho_{d}}{\rho_{w}+\rho_{d}(R_{c}-1)}$ (6)

$\approx\frac{R_{s}}{R_{c}}\mathrm{if}\rho_{d}\approx\rho_{w}.$ (7)

Since $\rho_{d}$ is restricted to be between the density of adipose and muscle tissue ($\sim0.9-1.06$ g mL^-1^ ), setting it equal to the density of water makes an error of at most 7% in the expansion ratio. The expansion ratio axis of Fig 2d is calculated with Eq (7) with $\rho_{d}=\rho_{w}$.

### 9.6 Computed Tomography

Computed Tomography (CT) scanning was performed in the UCLA Translational Research Imaging Center (TRIC) with a Siemens Somatom Definition 64 Dual Source Scanner for three different experiments (1 fast timescale, and 2 long timescale). For each experiment, eight tumescent injections were made in the abdomen of an anesthetized adult Yucatán pig, 2 each of 2.5 mL, 5 mL, 10 mL, and 20 mL physiological saline solution containing 20 mg mL^-1^ iodine contrast (6 mL Omnipaque 350 diluted to 100 mL). CT scans (resolution of $1\times1\times1$ mm) were made every 5-10 min for 70 min for the fast-time-scale experiment, or at $t=0, 0.5, 1, 2, 3, 4, 5, 6, 7$ hr for the two long-time-scale experiments. In the second long-time-scale experiment, epinephrine was added to the tumescent fluid at a ratio of 1:100000. Breath holds were administered during all scans.

Images were analyzed with OsiriX Lite DICOM viewer and analysis software. The attenuation of the pure tumescent solution (with contrast) was found to be 500 HU by imaging a syringe containing the fluid and averaging over its volume. The value in subcutaneous tissue was extracted by averaging over a region of normal, non-tumesced tissue and found to be -90 HU . With these values we can estimate the average expansion ratio with linear interpolation. If the fraction of tumescent fluid in a pixel is $f$, then the pixel value $v$ should be $v=-90(1-f)+500f$, with expansion ratio equal to $1/(1-f)=590/(500-v)$. For the fast timescale experiment, regions of interest were defined around the tumescent volumes by manually circumscribing their cross sectional area slice by slice. The software calculated the volume and mean pixel value within each volume, plotted in Figs 3b and 3c respectively. For the long-time-scale experiments however, such a procedure was not possible because the tumescent volumes became poorly defined as time progressed. Instead, lineouts through the center of the tumescent region as viewed from a coronal plane were extracted in maximum intensity projection (MIP) mode. The raw images are presented in Figs 5a and 5b, with corresponding lineouts in Fig 5c. Peak values of each lineout are plotted over time in Fig 5d.

### 9.7 3D Scanning

We used a Scannify 3D scanner (Fuel3D, Greenville, NC, USA) to capture the skin profile above a tumescent injection. The scanner works by taking standard 2D images from two cameras about a foot apart, with flashes originating from three different positions, and processing them with a cloud-based proprietary algorithm to reconstruct the 3-dimensional data with a manufacturer-specified resolution of 350 $\mu$m. All images for a single reconstruction are taken within a 0.1 s, which is fast compared to the dynamics we are interested in. After each scan there is a cool down time for the flash bulbs, which limits the minimum time between scans to about 30 s. We attached the scanner to a tripod that held it about 15 in. away from the region of interest, and controlled it from a laptop connected with USB. Since the scanner was sensitive to fur on the pig, we shaved the relevant area. To facilitate aligning the scans in post-processing, we placed hemispherical rubber stickers (about 1/4 in. diameter) on the pig’s skin as 3D markers. They were far enough from the injection site so that the skin profile did not change underneath them throughout the experiment. We made a 20 mL tumescent injection and took scans at $t=-0.5, 0, 0.7, 1.1, 2.0, 3.3, 6.5, 13.1, and 19.3$ min from the time of injection, defined to be just before $t=0$. Using the Fuel3D Studio software that came with the scanner, we cropped (in 3D) the region of interest from each scan, and exported an obj file containing a list of X, Y, Z coordinates laying on the pig-skin surface (see Fig 4a).

A Python script read in the obj files, aligned all the images to the one taken before the injection by minimizing the difference between the edges of the scans (where the swelling did not effect the skin profile), and rotated the coordinate system so that the tumescent expansion was directed along the Z direction. The direction of the X and Y axes were arbitrarily chosen. We verified proper alignment by taking lineouts of the skin profiles along the edges of the scans and making sure they overlapped. Lineouts of the intersection of the skin surface with the X=0 and Y=0 planes over time are shown in Figs 4b and 4c. The difference between the skin profiles after injection with the one before injection is a measure of the swelling over the X-Y plane, and has an appearance similar to a 2D Gaussian (see contour plots of Fig 4e and 4f). We define the tumescent height to be the maximum difference, and calculate the half-height area by counting the X-Y coordinate pairs whose corresponding Z value is greater than half the height. The tumescent volume height and half-height area are plotted against time in Fig 4d.
